# Elevated circulating monocytes and monocyte activation in COVID-19 convalescent individuals

**DOI:** 10.3389/fimmu.2023.1151780

**Published:** 2023-04-03

**Authors:** Juwon Park, Logan S. Dean, Boonyanudh Jiyarom, Louie Mar Gangcuangco, Parthav Shah, Thomas Awamura, Lauren L. Ching, Vivek R. Nerurkar, Dominic C. Chow, Fritzie Igno, Cecilia M. Shikuma, Gehan Devendra

**Affiliations:** ^1^ Hawaii Center for AIDS, John A. Burns School of Medicine, University of Hawai’i at Manoa, Honolulu, HI, United States; ^2^ Department of Tropical Medicine, Medical Microbiology, and Pharmacology, John A. Burns School Medicine, University of Hawai’i at Manoa, Honolulu, HI, United States; ^3^ Department of Medicine, John A. Burns School of Medicine, University of Hawai’i at Manoa, Honolulu, HI, United States; ^4^ John A. Burns School of Medicine, University of Hawai'i at Manoa, Honolulu, HI, United States; ^5^ Department of Pulmonary and Critical Care, Queen’s Medical Center, Honolulu, HI, United States

**Keywords:** SARS-CoV-2, Long-COVID, post-acute sequalae of SARS-CoV-2 infection, pulmonary sequelae, monocytes, CD169

## Abstract

**Background:**

Monocytes and macrophages play a pivotal role in inflammation during acute SARS-CoV-2 infection. However, their contribution to the development of post-acute sequelae of SARS-CoV-2 infection (PASC) are not fully elucidated.

**Methods:**

A cross-sectional study was conducted comparing plasma cytokine and monocyte levels among three groups: participants with pulmonary PASC (PPASC) with a reduced predicted diffusing capacity for carbon monoxide [DLCOc, <80%; (PG)]; fully recovered from SARS-CoV-2 with no residual symptoms (recovered group, RG); and negative for SARS-CoV-2 (negative group, NG). The expressions of cytokines were measured in plasma of study cohort by Luminex assay. The percentages and numbers of monocyte subsets (classical, intermediate, and non-classical monocytes) and monocyte activation (defined by CD169 expression) were analyzed using flow cytometry analysis of peripheral blood mononuclear cells.

**Results:**

Plasma IL-1Ra levels were elevated but FGF levels were reduced in PG compared to NG. Circulating monocytes and three subsets were significantly higher in PG and RG compared to NG. PG and RG exhibited *higher levels of* CD169^+^ monocyte counts and *higher* CD169 expression was detected in intermediate and non-classical monocytes from RG and PG than that found in NG. Further correlation analysis with CD169^+^ monocyte subsets revealed that CD169^+^ intermediate monocytes negatively correlated with DLCOc%, and CD169^+^ non-classical monocytes positively correlated with IL-1α, IL-1β, MIP-1α, Eotaxin, and IFN-γ.

**Conclusion:**

This study present evidence that COVID convalescents exhibit monocyte alteration beyond the acute COVID-19 infection period even in convalescents with no residual symptoms. Further, the results suggest that monocyte alteration and increased activated monocyte subsets may impact pulmonary function in COVID-19 convalescents. This observation will aid in understanding the immunopathologic feature of pulmonary PASC development, resolution, and subsequent therapeutic interventions.

## Introduction

It is estimated that one-third of patients infected with severe acute respiratory syndrome coronavirus 2 (SARS-CoV-2) who develop coronavirus-19 (1) continue to experience residual symptoms, collectively referred to as ‘Long-COVID’ or ‘post-acute sequelae of SARS-CoV-2 infection’ (PASC) (2). PASC symptoms are highly heterogeneous with a wide range of presentations, including fatigue, dyspnea, sleep disorders, anxiety, and loss of memory and/or concentration. Among individuals with PASC, pulmonary complications, such as persistent dyspnea and chronic cough are common (3, 4). The pathophysiology of COVID-19 is complex and appears to involve multiple inflammatory and immunological pathways (5). Studies have shown that COVID-19 patients display high systemic levels of cytokines and profound immune cell dysregulation that correlates with disease severity (6, 7).

Monocytes and macrophages are essential immune cells involved in host immunity and tissue homeostasis (8–10). These cells also possess inflammatory (11) and tissue-repairing capabilities and thus actively participate in all phases of the inflammatory response. Monocytes can be activated by infection and/or inflammatory conditions, leading to differentiation and polarization into macrophages with pro-inflammatory phenotypes (9, 12, 13). High monocyte count and activated monocyte phenotype have been linked to various pathological conditions (13–17). During SARS-CoV-2 infection, elevated peripheral monocyte levels and altered phenotype were observed in patients (14, 15, 18). Analysis of circulating monocytes has shown to predict disease severity and mortality in COVID-19 (19, 20). A comprehensive analysis of immune cells revealed long-term perturbations of innate and adaptive immune populations that persisted at least 6 months after SARS-CoV-2 infection (21). COVID-19 convalescents with prolonged symptoms displayed highly activated myeloid cells, lacked naïve T and B cells, and exhibited elevated type I and III interferon levels (22). A recent study found that intermediate (CD14^+^CD16^+^) and non-classical monocytes (CD14^lo^CD16^+^) were significantly elevated in PASC patients. Furthermore, SARS-CoV-2 S1 protein was detected in non-classical monocytes, but not classical and intermediate monocytes in PASC patients, suggesting that non-classical monocytes may contribute to inflammation in PASC (23). Although our understanding of innate immunity underlying the pathophysiology of PASC is evolving, a detailed understanding of monocyte response in individuals with pulmonary PASC (PPASC) remain unclear. Given that blood monocytes provide a window into the systemic immune response, reflecting the risk of potential complications after recovery from acute infection, it is important to characterize these monocyte populations to gain insight into the role that monocyte dysregulation plays in PPASC.

In this study, we analyzed circulating monocytes and plasma cytokine expression in COVID-19 convalescents, comparing them to uninfected individuals. We further assessed the relationship between these parameters and quantitative measures of lung function. We found that COVID-19 convalescents, regardless of residual pulmonary symptoms, displayed increased monocyte levels and had an activated phenotype, defined as CD169^+^ cells. Moreover, the percentages and numbers of CD169^+^ monocyte subsets were associated with the diffusing capacity of the lungs for carbon monoxide (DLCOc)% and proinflammatory cytokines, suggesting that alterations in monocyte subset activation may contribute to the development of chronic lung sequelae in individuals after resolution of COVID-19 infection.

## Methods

### Study cohort and selection of participants

This cross-sectional study investigated PASC complications among individuals living in Hawaii. Participants with PASC were recruited from the Post-COVID Recovery and Care Clinic of an academic tertiary care hospital (Queen’s Medical Center, Honolulu, Hawaii) between September 2020 and Mar 2021, prior to the detection of Omicron variants in Hawaii. Participants were grouped into the following: A) individuals who reported persistent pulmonary symptoms (dyspnea, fatigue, cough, or shortness of breath) beyond 30 days after COVID-19 infection with reduced diffusion capacity for carbon monoxide (corrected for hemoglobin-DLCOc, <80%) by pulmonary function test (pulmonary PASC group [PG]; n=11); B) individuals who have fully recovered from SARS-CoV-2 infection with no residual symptoms >30 days after acute infection (recovered group [RG]; n=10); and C) individuals confirmed to have not contracted COVID-19 using negative SARS-CoV-2 antibody test (negative group [NG], n=10). The PG and RG groups had documented positive SARS-CoV-2 by polymerase chain reaction (PCR) and a replicated SARS-CoV-2 IgG antibody test. The study was approved by the Queens Medical Center Institutional Review Committee with the University of Hawaii IRB ceding authority (24: RA-2020-053).

### Pulmonary function tests

Pulmonary function testing (PFT) was performed on individuals PPASC. All PG participants underwent PFTs (Vyaire) with the measurements of forced vital capacity (FVC), forced expiratory volume in 1 second (FEV1), total lung capacity (TLC), and DLCOc% interpreted in accordance with European Respiratory Society (ERS)/American Thoracic Society (ATS) guidelines (24).

### Plasma and peripheral blood mononuclear cells isolation 

Whole blood was collected from study participants in ethylene diamine tetra-acetic acid (EDTA) tubes (BD, Vacutainer) by venipuncture and processed based on a well-established method (25). In brief, whole blood was centrifuged, plasma removed and cryopreserved at −80°C until downstream analysis. Remaining venous blood was diluted with an equal volume of phosphate buffered saline (PBS) and layered on top of Ficoll-Paque Plus (GE Healthcare Biosciences, Piscataway, NJ) following the manufacturer’s protocol. PBMC were separated by centrifugation at 400 × g for 30 minutes at room temperature (RT). PBMC were collected from the buffy coat, red blood cells lysed, and then washed twice in PBS supplemented with 1% fetal bovine serum (FBS). Cells were then counted, viability determined, and cryopreserved until further analysis.

### Multiplex cytokine analysis

Plasma was thawed and prepared following the manufacturer’s guidelines for each kit. All samples were run in duplicate in a single plate per panel. Biomarkers to assess inflammation (IFN-γ, IL-13, IL-1α, IL-8, IL-1β, IL-1Ra, TNF-α), leukocyte chemotaxis (Eotaxin, MCP-1, MIP-3α, MIP-1α, RANTES/CCL5), and tissue remodeling/fibrosis (PDGF-AA, PDGF-AA/BB, FGF, VEGF, TGF-α) were measured using the R&D System™ Human XL Cytokine Discovery Premixed Kit. Data were acquired on a MAGPIX^®^ Instrument (Luminex Corporation, Austin, TX). Data analysis was done using GraphPad Prism 9. Net median fluorescent intensity (MFI) was calculated (MFI value minus background value) and the average net MFI of duplicate samples was determined.

### Flow cytometric analysis

Cryopreserved PBMC were thawed and washed twice in PBS. Typically, cells were incubated with LIVE/DEAD™ Fixable Yellow Dead Cell Stain (Invitrogen, 1:1000) at 4°C for 30 minutes, followed by addition of Human TruStain FcX (BioLegend, San Diego, CA, 1:200) in hanks balanced salt solution (HBSS) supplemented with 1% bovine serum albumin (BSA) (flow buffer) at room temperature (RT) for 15 minutes. Subsequently, cells were stained with the titrated fluorophore conjugated extracellular antibodies; CD45-BV711 (BD Biosciences, East Rutherford, NJ), CD11b-PE-Cy-7 (BioLegend, San Diego, CA), CD14-BV605 (BioLegend, San Diego, CA), CD16-BV421 (BioLegend, San Diego, CA), CD169-APC (Biolegend, San Diego, CA) at RT for 30 minutes and then washed twice with ice-cold flow buffer. Samples were then washed twice with ice-cold flow buffer and resuspended to 800 ɱL of flow buffer for acquisition. Samples were acquired with identical voltage settings on a LSR Fortessa (BD Sciences, East Rutherford, NJ) with approximately 1.0x10^9^ events collected per sample. 10 uL of AccuCheck Counting Beads (Life Technologies, Carlsbad, CA) were added prior to acquisition for calculation of absolute cell counts. Compensation beads (Invitrogen, Waltham, MA) were prepared for accurate compensation controls. Data was analyzed using FlowJo (Treestar, Ashland, OR) software and absolute cell counts were determined according to manufacturer protocols.

### Statistics

A cross-sectional study comparing PG, RG, and NG participants was undertaken. Flow cytometry results between the groups were compared using Mann Whitney-U test. Patient characteristics between groups were compared using Chi-squared test, Fisher’s exact test, or Mann Whitney-U test, as appropriate. Correlation between monocyte subsets, CD169^+^ monocyte subsets, and inflammatory markers were analyzed using Spearman correlation. P-value < 0.05 was considered statistically significant for all tests. Statistical analyses were performed using IBM SPSS version 28 (Chicago, IL) and GraphPad Prism 9. (Graph Pad, San Diego, CA).

## Results

### Study cohort description

The median age of the participants was 53, 54, and 55 years for PG (n=11), RG (n=10), and NG (n=10), respectively. 18.2% of individuals with PPASC had respiratory history (asthma or COPD). Higher rates of hospital admission were seen among PG compared with RG (36% vs 0%, P = 0.015). Among the hospitalized patients (n=4), one patient was admitted to the medical intensive care unit (ICU) due to increasing oxygen demand and eventual mechanical ventilatory support. No significant differences in pre-existing conditions, body mass index (BMI), and smoking prevalence between PG and RG were seen. Overall, 68.8% of the participants had received a SARS-CoV-2 vaccine at the time of enrollment. Individuals with PPASC experienced prolonged pulmonary complications including, dyspnea, fatigue, cough, and shortness of breath; 45.5% of them had at least two pulmonary symptoms. Those with PPASC reported symptoms lasting for a median duration of six months from post COVID-19 infection. All individuals from RG resolved symptoms within 4 weeks after disease onset and reported no symptoms at the time of sample collection. The baseline participant characteristics are displayed in **Table 1**.

**Table 1:** Characteristics of study participants.

	PG (N=11)	RG (N=10)	NG (N=10)	P-value
Age (years)	53 [44, 58]	54 [46.2, 59.2]	55 [44, 58.2]	0.953
Male Gender	8 (72.7%)	6 (60%)	6 (60%)	0.778
Months post-COVID infection	6 [4, 12]	5 [1.5, 9.5]*	--	0.515
Body Mass Index (kg/m^2^)	30.8 [24.3, 35.1]	26.9 [22.0, 34.6]	No data	0.676
Received vaccination for COVID-19	9 (81.8%)	5 (50%)	8 (88.9%)*	0.116
Hospital admission (n, %)**	4 (36.3%)	0	0	**0.015**
Race				
White/Caucasian	3 (27.3%)	1 (10%)	5 (50%)	0.160
Asian	4 (36.4%)	4 (40%)	5 (50%)	
Native Hawaiian/Pacific Islander	3 (27.3%)	2 (20%)	0	
Other	1 (9.0%)	3 (30%)	0	
Co-morbidities				
Asthma	1 (9.1%)	1 (10%)	1 (10%)	0.997
COPD	1 (9.1%)	0	0	0.391
Hypertension	3 (27.3%)	2 (20%)	1 (10%)	0.605
Diabetes	2 (18.2%)	1 (10%)	1 (10%)	0.809
Smoking history (past/current)	4 (36.4%)	2 (20%)	0	0.109
Current alcohol use	3 (27.3%)	5 (50%)	0	**0.038**

P-value calculated using Kruskal-Wallis test, Chi-square test, or Fisher’s exact test.

*1 missing data.

**One patient was admitted to the medical ICU for mechanical ventilatory support.

Bold indicates statistical significance.

### Soluble biomarker levels in COVID-19 convalescents

**Figure 1:**
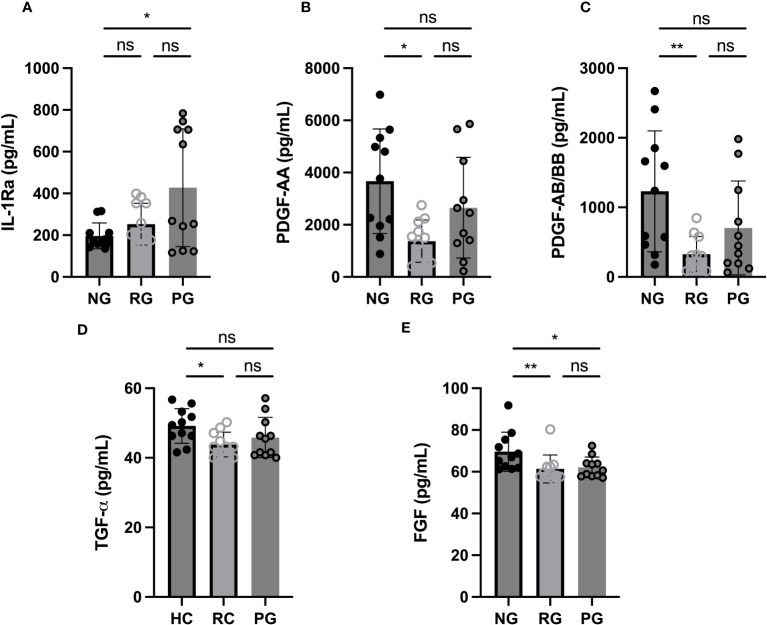
Plasma cytokine levels among groups. **(A)** IL-1Ra, **(B)** PDGF-AA, **(C)** PDGF-AB/BB, **(D)** TGF-α, and **(E)** FGF levels were measured in plasma collected from NG, RG, and PG. The data are represented as the box and scatter plots with each circle representing a single individual. P values were calculated using the Mann–Whitney U-test. *p < 0.05, **p < 0.01, ns, non-significant.

To examine blood biomarkers associated with PPASC, we assessed 17 analytes in the plasma of participants from the NG, RG, and PG by Luminex assay and compared the plasma concentration of analytes among the groups. Analytes included cytokines associated with “cytokine storm”; IL-1α, IL-1β, IL-1Ra, IL-8, IL-13, and TNF-α. “leukocyte chemotaxis”; Eotaxin, MCP-1, MIP-3α, MIP-1α, Fractalkine, IP-10, MCP-3, and, RANTES.), “tissue remodeling”; PDGF-AA, PDGF-AA/BB, FGF, VEGF, and TGF-α. The IL-1Ra was elevated (2.2 fold higher) in the PG compared to NG, and its’ level trended higher in PG as compared to RG (**Figure 1A**). PDGF-AA, PDGF-AB, and TGF-α were decreased in the RG, compared to the NG, and their levels tended to be lower in the PG (**Figures 1B–D**). FGF remained significantly lower in the RG and PG compared to NG (**Figure 1E**). There was no difference observed in 13 analytes among three groups (**Supplementary Figure 1**).

### COVID-19 convalescents display elevated circulating monocytes

**Figure 2:**
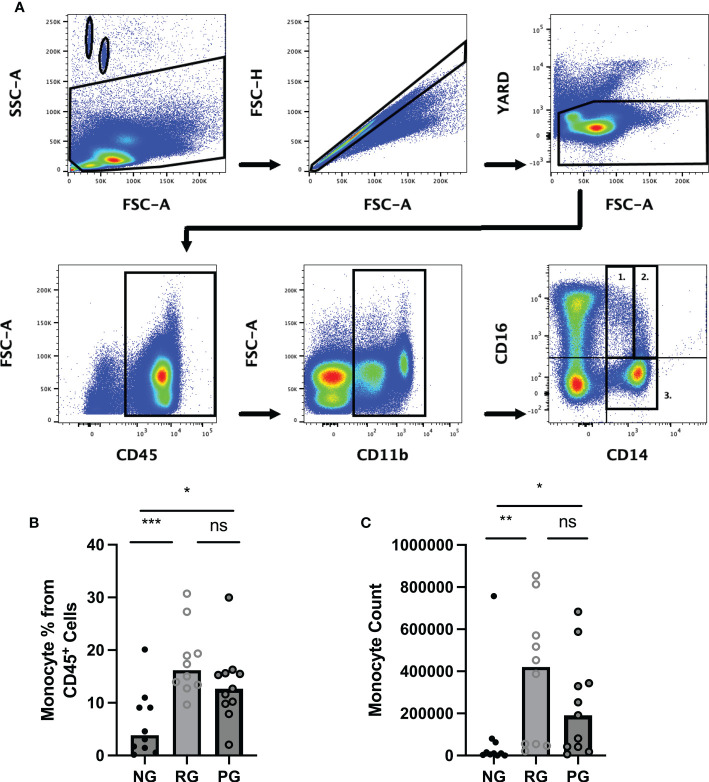
Comparison of circulating monocyte levels among the groups. Representative flow cytometry gating strategy for identification of monocytes and monocyte subsets in PBMC from NG, RG, and PG groups. Lymphocytes and monocytes were selected using CD45^+^ followed by gating for CD11b^+^ cells. (A1) non-classical (CD14^lo^/CD16^+^), (A2) intermediate (CD14^+^/CD16^+^, (A3) Classical (CD14^+^/CD16^-^), and **(B)** Total monocyte percentages as a proportion of total identified CD45^+^ cells in NG, RG, and PG groups. **(C)** Total monocyte counts in NG, RG, and PG groups. Mann-Whitney-U Test *p < 0.05, **p < 0.01, ***p < 0.001, ns, non-significant.

**Figure 3:**
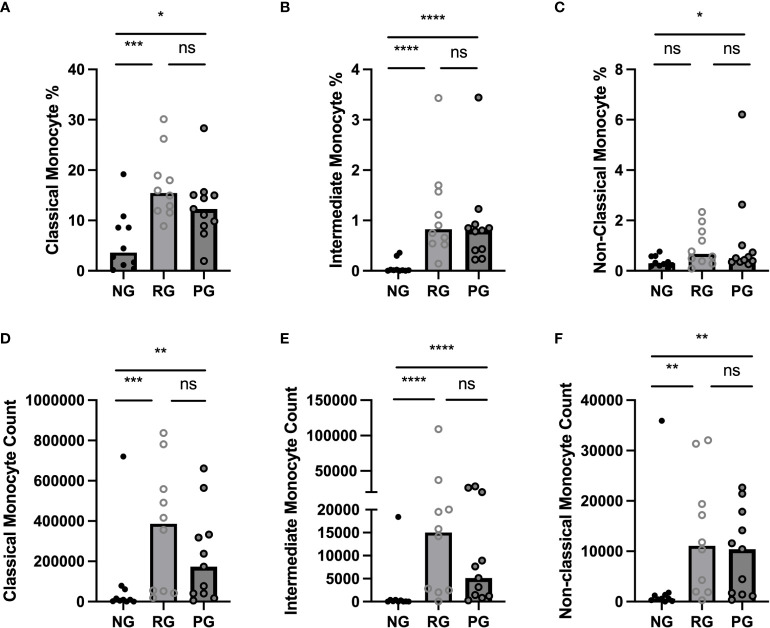
Comparison of circulating monocyte subsets among the groups. **(A)** Classical monocyte percentage, **(B)** Intermediate monocyte percentage, **(C)** Non-Classical monocyte percentage, as a proportion of total identified CD45^+^ cells in NG, RG, and PG groups. **(D)** Classical monocyte counts, **(E)** Intermediate monocyte counts, and **(F)** Non-Classical monocyte in NG, RG, and PG groups. Mann-Whitney-U Test *p < 0.05, **p < 0.01, ***p < 0.001, ****p < 0.0001, ns, non-significant.

To determine the impact of long-term consequence of COVID-19 on blood monocytes and monocyte alteration in PPASC, PBMC cells isolated from NG (n=10), RG (n=10), and PG (n=11) cohorts were analyzed by flow cytometry. A representative gating strategy for identifying monocytes within the PBMC fractions from three groups was shown in **Figure 2A**. Monocyte subsets; classical (CD14^+^ CD16^-^), intermediate (CD14^+^ CD16^+^), or non-classical (CD14^lo^ CD16^+^) were defined by CD14 and CD16 surface levels within the monocytes (**Figure 2A**). We found both the percentages and numbers of total circulating monocytes were higher in the COVID-19 convalescents (PG and RG) than NG (**Figures 2B, C**). In comparison to NG, both the percentage and number of classical and intermediate monocytes were significantly increased in PG and RG (**Figures 3A, B, D, E**). Also, the percentage and number of non-classical monocytes were significantly increased in PG, but only non-classical monocyte numbers were increased in RG, compared to NG (**Figures 3C, F**). However, we did not observe any significant difference between PG and RG in three monocyte subsets percentages and numbers (**Figure 3**). Altogether, these observations suggest that circulating monocyte levels remain elevated for several months after SARS-CoV-2 infection, even convalescents who have no residual symptom.

### CD169^+^ monocytes in COVID-19 convalescents

**Figure 4:**
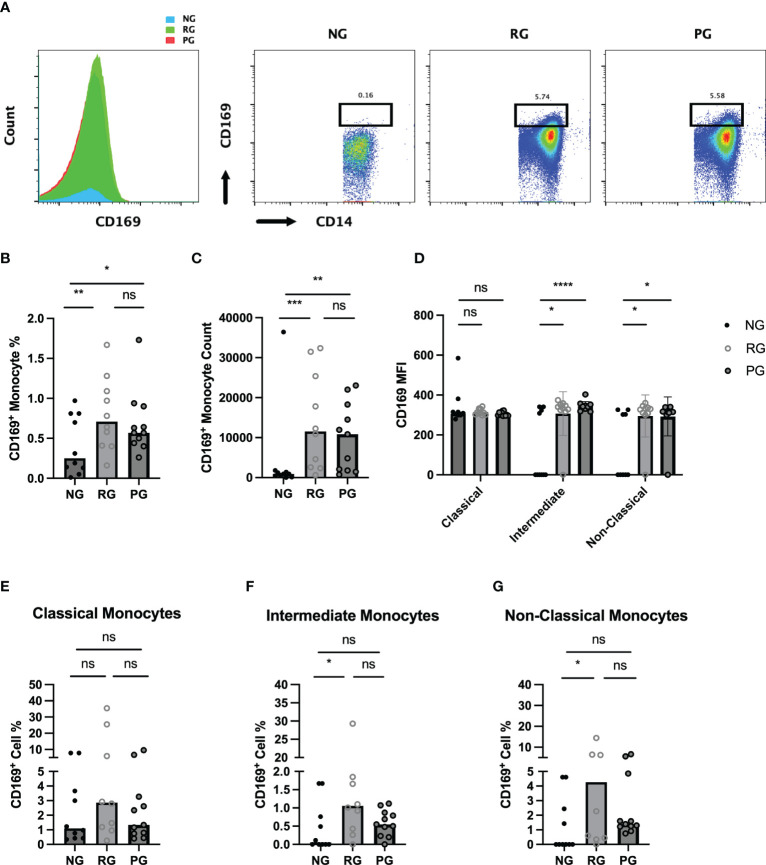
Characterization of circulating CD169^+^ monocytes in NG, RG, and PG groups. Representative histogram and dot plot of CD169^+^/CD14^+^ monocytes in NG, RG, and PG groups. Percentage of total CD169^+^ monocytes from total CD14^+^ monocytes is shown above the gate **(B).** Total CD169^+^ monocyte percentage from CD45^+^ cells, **(C)** CD169^+^ monocyte, **(D)** MFI of CD169 on monocyte subsets in NG, RG, and PG. The percentage of CD169^+^ cells identified in **(E)** classical monocytes, **(F)** intermediate monocytes, and **(G)** non-classical monocytes within NG, RG, and PG groups. Mann-Whitney-U Test *p < 0.05, **p < 0.01, ***p < 0.001, ****p < 0.0001, ns, non-significant.

CD169, a type I interferon-inducible receptor, is expressed on monocytes and macrophages (26–28). CD169^+^ monocytes and macrophages have been thought to be important players in inflammatory response of inflammatory and autoimmune diseases (29–31). Monocytes from COVID-19 patients had increased CD169 levels during acute SARS-CoV2 infection and monocyte CD169 was identified as a biomarker in early COVID-19 infection (27). To further investigate difference in monocyte activation among the groups, we analyzed CD169 expression in monocytes. When monocytes were stratified based on CD169 expression, the percentage of CD169^+^ monocytes were significantly higher in the PG and RG than in the NG (**Figure 4A**). Also, CD169^+^ monocyte numbers were significantly increased in the PG and RG, compared to NG (**Figure 4B**). The MFI of CD169 on classical monocytes did not differ among the groups. However, CD169 MFI of intermediate and non-classical monocytes in PG and RG was significantly higher than those in NG (**Figure 4C**). Interestingly, when the percentage of CD169^+^ cells was examined in the three groups in each respective monocyte population, no difference was observed in classical monocytes (**Figure 4D**). Significant increases in CD169^+^ percentages were observed in intermediate and non-classical monocytes, only between RG and NG, with no difference between PG and NG, or PG and RG observed (**Figures 4E–G**). These data indicate that circulating monocytes from COVID-19 convalescents remain increased and display a higher CD169 expression.

### The relationship between CD169^+^ monocytes and lung function in PPASC

**Figure 5:**
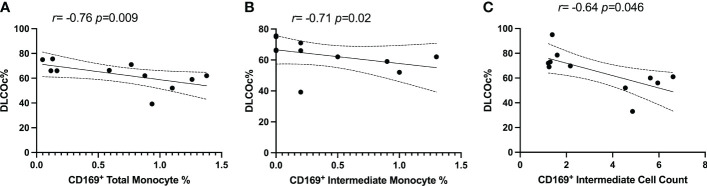
Spearman correlation between DLCOc% and **(A)** percentage of CD169^+^ total monocytes, **(B)** percentage of CD169^+^ intermediate monocytes, and **(C)** CD169^+^ intermediate monocyte count in PG.

PG participants had PFTs performed during their period of prolonged respiratory symptoms. 18.2% of participants reported pre-existing pulmonary conditions prior to infection with SARS-CoV-2 (**Table 1**). We explored the relationship between the percentages and counts of CD169^+^ monocyte with DLCOc%. We found negative correlations between CD169^+^ monocytes and CD169^+^ intermediate monocytes percentages and CD169^+^ intermediate monocyte counts (r=-0.758; P=0.009, r=-0.71; P=0.02, r=-0.69; P=0.051, respectively) and DLCOc% in PG (**Figures 5A–C**). Taken together, these results suggested that elevated CD169 expression in monocytes may serve as biomarker for determining lung function in PPASC.

### Monocyte relationship with systemic levels of cytokines in PPASC

**Figure 6:**
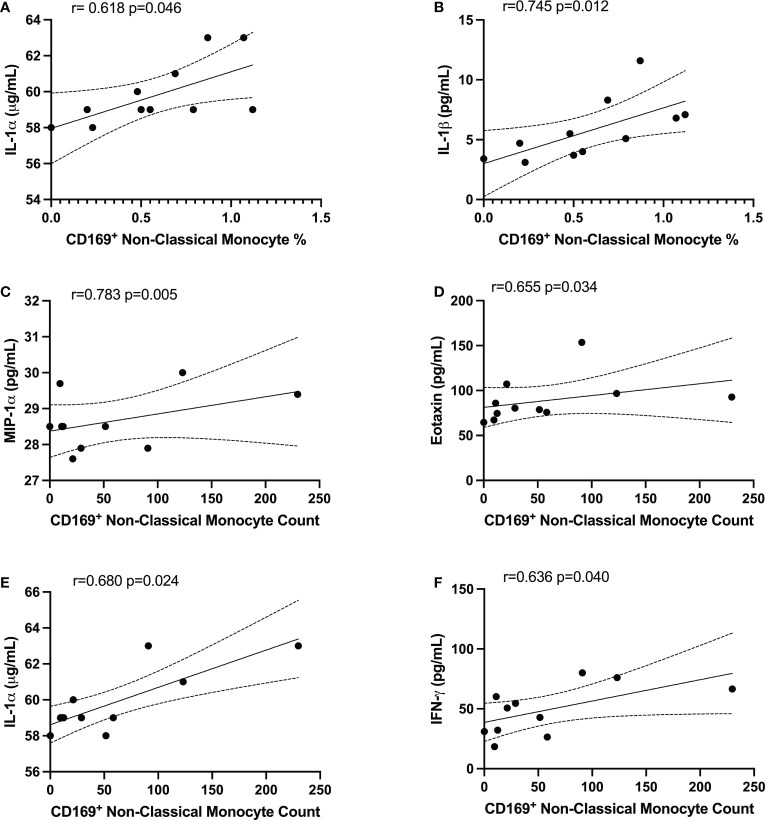
CD169^+^ non-classical monocytes were associated with IL-1α, IL-1β, MIP-1α, Eotaxin, and IFNγ in PG. Spearman correlation between the percentage of non-classical CD169^+^ monocytes and **(A)** IL-1α and **(B)** IL-1β. Spearman correlation between CD169^+^ non-classical monocyte count and **(C)** MIP-1α, **(D)** Eotaxin, **(E)** IL-1α, and **(F)** IFNγ.

In order to determine whether the changes in monocytes correlated with cytokine expression in PPASC, we performed spearman rank correlation to assess associations between cytokines and monocyte subset counts and percentages. In **Table 2**, monocyte parameters (monocyte subsets and CD169^+^ total monocyte and monocyte subsets) showed a positive correlation with cytokines (VEGF, IL-8, IL-1α, IL-1β, PDGF-AA, PDGF-AB/BB, Eotaxin, MIP-1α, MCP-1, and IFN-γ). VEGF levels were positively associated with CD169^+^ total monocyte counts and non-classical monocyte percentages. The percentage of intermediate monocytes was positively associated with IL-1α, IL-1β, MIP-1α, PDGF-AA, and PDGF-AB/BB cytokines, but negatively associated with FGF. Eotaxin, MIP-1α, and VEGF were positively associated with intermediate monocyte counts, while MCP-1 was associated with the CD169^+^ percentage of intermediate monocytes. IL-1α and IL-1β were positively correlated with the percentage of CD169^+^ non-classical monocytes. IL-1α, MIP-1α, IFN-γ, Eotaxin, and PDFG-AA were positively correlated with CD169^+^ non-classical monocyte counts (**Table 2** and **Figure 6**). Altogether, these results suggested that monocyte subsets and the cells with CD169 upregulation were associated with a proinflammatory cytokine environment in PPASC.

**Table 2:** Spearman correlations of circulating molecules and monocyte populations in PG.

Parameter	Analyte	R-value	P-value
Total CD169^+^ Monocyte Count	VEGF	0.84	0.002
Intermediate Monocyte % of CD45^+^ Cells	MIP-1α	0.611	0.05
	IL-1α	0.709	0.017
	IL-1β	0.8	0.005
	PDGF-AA	0.764	0.009
	PDGF-AB/BB	0.782	0.006
	FGF	-0.627	0.044
Intermediate Monocyte Count	Eotaxin	0.627	0.044
	MIP-1α	0.63	0.042
	VEGF	0.736	0.013
Non-classical Monocyte % of CD45^+^ cells	IL-8	0.747	0.04
	MCP-1	-0.709	0.018
Non-classical Monocyte Count	VEGF	0.836	0.002
CD169^+^ % of Intermediate Monocytes	MCP-1	0.691	0.022
CD169^+^ Non-classical Monocyte % of CD45^+^ cells	IL-1α	0.618	0.047
	IL-1β	0.745	0.011
CD169^+^ Non-classical Monocyte Count	IFN-γ	0.636	0.04
	Eotaxin	0.655	0.034
	PDGF-AA	0.618	0.048
	IL-1 α	0.68	0.024
	MIP-1α	0.783	0.006

## Discussion

In this study, we observed that COVID-19 convalescents with pulmonary PASC displayed altered circulating monocyte levels and activation, which may last several months after infection. Interestingly, monocyte alterations were also observed in individuals whose symptoms had resolved completely. These findings highlight that COVID-19 convalescents exhibit monocyte dysregulation beyond the resolution of initial infection.

Monocytes have begun to emerge as key cellular modulators of COVID-19 pathophysiology. During acute SARS-CoV-2 infection, monocytes are dysregulated, exhibiting aberrant functions and mediation of the cytokine storm associated with severe COVID-19. Zhou et al. (11) found elevated levels of CD14^+^CD16^+^ monocytes in ICU COVID-19 patients, compared to non-ICU patients and healthy controls. Indeed, granulocyte-macrophage-colony stimulating factor (GM-CSF) and IL-6 expressing CD14^+^ monocytes were significantly increased in the ICU patients. Few studies have detailed the immune profiles in individuals with PASC despite accumulating evidence indicating that substantial perturbation of the innate immune system is presents in PASC. However, it is still largely unknown if immune cell perturbations contribute to the immunopathology of PPASC.

This is the first study, to our knowledge, to evaluate the associations of monocyte levels with lung function in a community-based cohort. In this study, we selected individuals with PPASC through a primary questionnaire and secondary evaluation of pulmonary function. This approach clarified the presence of pulmonary symptoms and may not discern individuals who had their symptoms overestimated based on the questionnaire. Studies demonstrate that pulmonary symptoms were present regardless of initial COVID severity, but patients with severe COVID-19 were more likely to have impairment of lung function (32, 33). Also, pulmonary symptoms were presented without impairment of lung function or cardiopulmonary exercise test among COVID-19 survivors (33). Also, monitoring of PFTs in severe COVID-19 survivors with lung abnormalities post discharge demonstrated significant pulmonary sequelae (34, 35). These study demonstrates that hospitalized COVID-19 survivors were more likely to have persistent pulmonary PASC symptoms compared with those without hospitalization. Hospitalized COVID-19 survivors tended to have reduced DLCOc% (61.98%) compared to non-hospitalized COVID-19 survivors (66.89%). A correlation analysis of CD169^+^ monocyte subsets and DLCOc% suggested that alteration of activated monocyte subsets may impact pulmonary function in COVID-19 convalescents. Nonetheless, we cannot exclude the possibility that this association may reflect other residual symptoms because a nonnegligible proportion of PPASC individuals also experienced various other symptoms.

The cytokine profile revealed no changes in major inflammatory cytokines between NG, RG, and PG. Similar observations in COVID-19 convalescents have been reported in another cross-sectional study. IL-1α, IL-1β, IL-8, IFN-γ, VEGF-A, and TNF-α had returned to normal levels 6 months after recovery, but IL-1Rα was still elevated in COVID-19 convalescents, compared to healthy controls (20). Another study showed that COVID-19 convalescents at 4 months post infection had higher levels of IFN-β, IFN-λ1, CXCL9, CXCL10, IL-8, and sTIM-3, regardless of symptoms, compared to uninfected controls. IFN-β and IFN-λ1 still remained elevated in COVID-19 convalescents with PASC but others’ expressions were reduced at month 8, compared to month 4 (22). Queiroz et al. (36) showed that IL-2, TNF-α, and IL-17 levels were higher in COVID-19 convalescents than acute COVID-19 patients. Also, individuals with PASC had higher levels of IL-17 and IL-2, but lower levels IL-10, IL-6, and IL-4 levels, compared individuals without sequelae. However, there was no significant difference in IL-6 levels between post-COVID-19 individuals with and without sequelae (36). Oher studies investigating immune features of COVID-19 convalescent trends observed elevated levels of IL-6 and IL-1β (20, 37, 38) in individuals with PASC. Notably, increased IL-1β, IL-6, and TNF levels were reported in association with PASC development in a large-scale cohort study (38). Interestingly, published scRNA-sequencing datasets generated from severe COVID-19 patients demonstrate increased transcript reads of IL-1β and TNF-α from bronchoalveolar lavage fluid (BALF) macrophages (38), supporting their hypothesis that proinflammatory reprogramming of lung macrophages and/or precursor monocytes may drive prolonged and exacerbated PASC symptomology.

Some discrepancies in the reported cytokine levels from PASC studies continue to generate questions regarding the importance of a heterogeneous multisystemic condition. One might speculate the varying windows of sample collection post-infection between various studies alters the detectable cytokine profiles. Another possibility is that COVID-19 convalescents are not classified into symptomatic and asymptomatic. In this case, less systemic inflammation is represented, but the respiratory environment may show distinct proinflammatory conditions in individuals with PPASC. Further analysis of cellular composition and cytokines in blood and BALF from individuals with PPASC over a longitudinal period is required to understand the dynamic features of respiratory and systemic immunity in PPASC during disease progression and resolution.

While no difference in major inflammatory cytokines between the three groups was observed in our study, correlations of CD169^+^ monocyte subsets with cytokines suggested that specific activated monocyte subsets produce high levels of proinflammatory cytokines in PPASC. Correlations of D-dimer with CD169^+^ non-classical monocytes observed herein were corroborated by the findings of Pandori et al. (39) in a cohort of individuals hospitalized for COVID-19 (39). Interestingly, their cohort did not display the increases in total monocyte populations in their hospitalized group but displayed decreases in non-classical monocyte percentages and steady levels of classical and intermediate monocyte percentages from total CD45^+^ cells in participants hospitalized for COVID-19 up to 90 days following admission. These trends suggest that decreased monocyte proportions are present during hospitalization from COVID-19, but COVID-19 convalescents demonstrated elevated monocyte levels, potentially in a dysregulated nature (20, 22, 23).

A recent publication analyzed soluble factors related to monocyte/macrophage dysregulation and SARS‐CoV‐2 spike (S1) protein in COVID-19 convalescents (40). They demonstrated that prolonged perturbations of IL-5 and IL-17F levels were observed in individuals with sequelae. Also, these individuals showed few significant correlations among tested cytokines, but this association was not evident in individuals without sequelae. Furthermore, higher circulating S1 levels were detected in individuals with sequelae, compared to individuals without sequelae. In line with their findings, persistent S1 protein were found significantly increased in non-classical monocytes in individuals with PASC up to 15 months post-infection (23), indicating the presence of replicating viral reservoirs in PASC. Further questions are raised as to whether monocyte subsets represent a key inflammatory driver of PPASC pathogenesis and what the consequences of viral reservoirs in non-classical monocytes are in PASC persistence. Further studies for monitoring monocyte and cytokine perturbations and viral reservoirs over time in a larger cohort warrants further investigation to identify a suitable biomarker for PPASC prognosis prediction and prognosis.

Limitations of this study include a small sample size and a lack of initial COVID-19 severity and medical history in comparison groups (NG and RG). Recent studies have demonstrated that female sex, age, and smoking are risk factors for PASC (41–43). However, the majority of our PPASC individuals were male (72.7%) and older, thus we did not have younger participants to stratify our analyses by age. Likely due to a small sample size, there were no differences in the level of monocytes or immunological parameters between males and females. Sample size was based on feasibility, rather than an objective estimation step driven by a hypothesis and our sample size was not adequately powered for multivariable analyses. Therefore, we cannot be certain if main risk factors for severe and PASC includes, age, sex, smoking, and comorbidities contribute to monocyte dysregulation in COVID-19 convalescents.

Our clinical data was also limited by chart review and/or patient recall. From our chart review of the PPASC patients (N=11), one patient received a combination of remdesivir, dexamethasone, and convalescent plasma; one patient received remdesivir and dexamethasone. Some patients could not recall whether they received any specific interventions for their acute COVID-19 episode. The length of post COVID-19 infection was variable, 1 to 10 months post-infection. Variability in time of sample collection may have influenced monocyte population characteristics. Due to the exploratory nature, the limited sample size, and variable sampling time, we acknowledge that a large sample size would have provided increased statistical power and more informed conclusions of monocyte roles in PPASC. In addition, a longitudinal evaluation of monocytes dynamics and the phenotypic changes after COVID-19 infection should be carried out to determine whether monocytes dysfunction is associated with the clinical outcome of respiratory failure. It would also be of clinical interest to track the perturbations of monocyte populations in relation to acute infection, COVID-19 disease context, and then into PPASC development or recovery.

In summary, our data indicate that systemic monocyte alteration continues within COVID-19 convalescents with pulmonary symptoms, which is also found in COVID-19 convalescents with no residual symptoms. Also, COVID-19 convalescents exhibit activated monocyte phenotypes, denoted by CD169 expression, and this activated phenotype is associated with poor lung function and increased proinflammatory cytokines. The drivers of PPASC pathogenesis require further investigation, but possibilities include high circulating monocyte levels, increased CD169^+^ intermediate and non-classical monocytes, and IL-1Ra expression. These observations will aid in informing the ongoing decipherment of the immunopathology that contributes to PPASC development, COVID-19 recovery, and subsequent therapeutic interventions.

## Data availability statement

The raw data supporting the conclusions of this article will be made available by the authors upon request. 

## Ethics statement

The study was approved by the Queens Medical Center Institutional Review Committee with the University of Hawaii IRB ceding authority (24: RA-2020-053). The patients/participants provided their written informed consent to participate in this study.

## Author contributions 

JP supervised and designed the experimental approaches. LD conducted and analyzed flow cytometry data. BJ collected and processed human blood samples and conducted the Luminex experiments and data analysis. GD analyzed PFT and clinical data collection. LG analyzed data and assisted in generating table. LC, VN, DC, and CS contributed to subject recruitment and sample collection. JP, LD, LG, and TA wrote the draft of the manuscript. PS, VN, DC, FI, CS, and GD revised the manuscript. All the authors assisted in editing, provided critical review, and approved the final version of the submission. All authors contributed to the article and approved the submitted version.

## Supplementary material

Supplementary Figure 1 Levels of plasma cytokine in NG, RG, and PG. **(A)** No differences in Eotaxin, MCP-1, MIP-3α, MIP-1α, IFNγ, IL-13, IL-1β, TNFα, VEGF, RANTES/CCL5, **(B)** IL-8 and IL-1α.
