# Supplementary figures and images for: Elevated circulating monocytes and monocyte activation in COVID-19 convalescent individuals
Source: Front Immunol. 2023 Apr 3;14:1151780. doi: 10.3389/fimmu.2023.1151780 (PMC10106598; doi:10.3389/fimmu.2023.1151780)

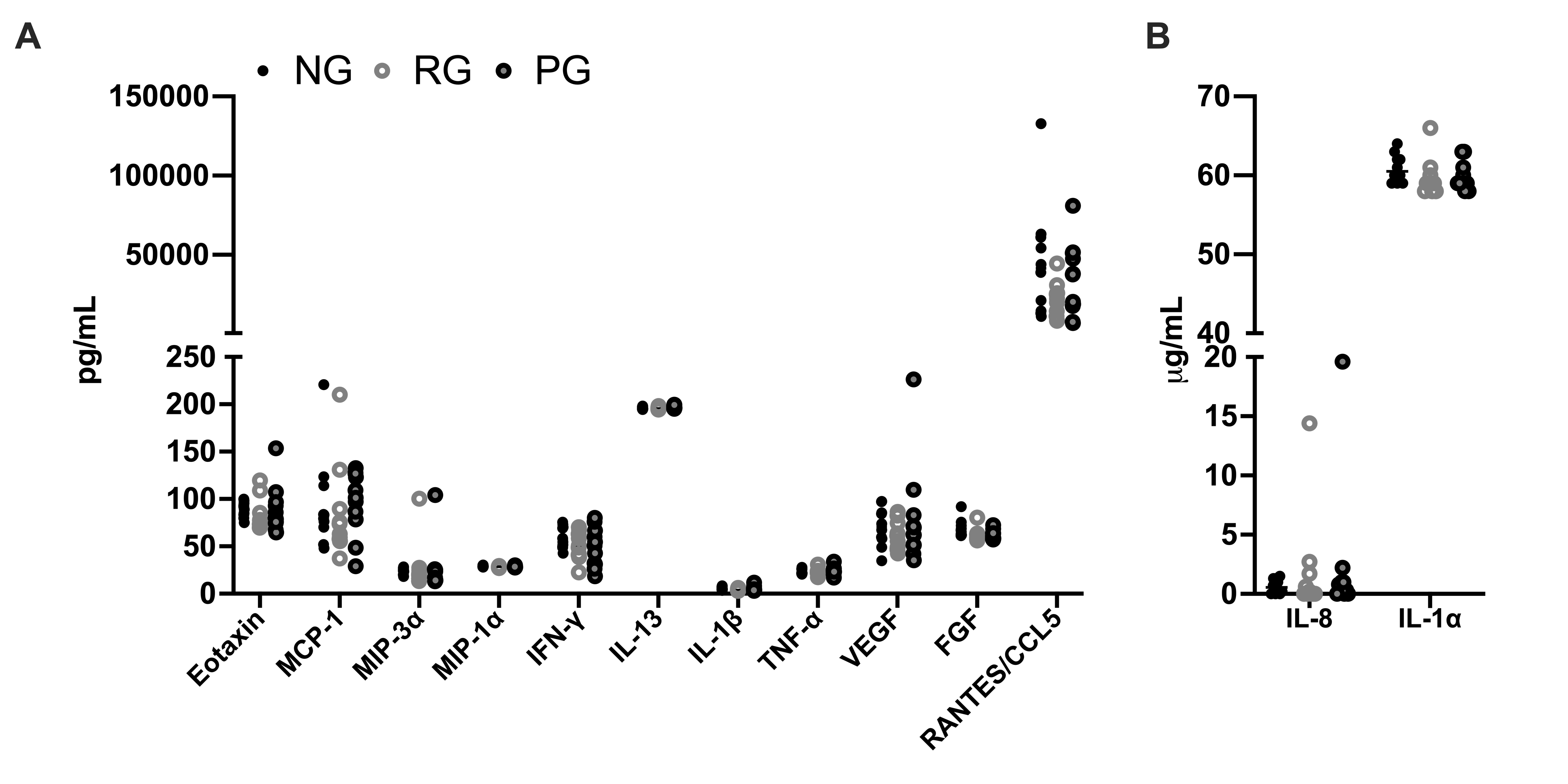

Supplement: Supplementary Figure 1 — Levels of plasma cytokine in NG, RG, and PG. (A) No differences in Eotaxin, MCP-1, MIP-3α, MIP-1α, IFNγ, IL-13, IL-1β, TNFα, VEGF, RANTES/CCL5, (B) IL-8 and IL-1α. [file Image_1.tiff]
